# A case of right middle lobectomy for primary lung cancer in a patient with heterotaxy syndrome

**DOI:** 10.1186/s44215-024-00177-z

**Published:** 2024-11-22

**Authors:** Ryo Demura, Kazuhiro Imai, Shinogu Takashima, Nobuyasu Kurihara, Shoji Kuriyama, Haruka Suzuki, Yuzu Harata, Yoshihiro Minamiya

**Affiliations:** Department of Thoracic Surgery, Akita University Graduate School of Medicine, 1-1-1 Hondo, Akita, 010-8543 Japan

**Keywords:** Primary lung cancer, Anomaly, Heterotaxy syndrome, Left isomerism

## Abstract

**Background:**

Anatomical abnormalities in the pulmonary vessels have long aroused great interest among thoracic surgeons, and numerous variations of pulmonary vessels have been reported. Heterotaxy syndrome is an anatomical abnormality in which typically asymmetrical organs, including the lungs, develop symmetrically. We report the case of a 71-year-old man with heterotaxy syndrome undergoing radical lobectomy in the treatment of non-small cell lung cancer.

**Case presentation:**

Computed tomography (CT) revealed an irregular nodule 25 mm in diameter in the right middle lobe. Two months later, at his first visit to our University Hospital, CT revealed a rapidly growing tumor 60 mm in diameter. In addition, three-dimensional (3D) CT revealed the upper and middle lobar bronchi forming a common trunk with the mediastinal type of the right pulmonary artery (PA). The patient underwent video-assisted right middle lobectomy + systematic complete hilar and mediastinal lymph node dissection. The interlobar fissure between the right upper and middle lobes was incomplete, and the common trunk formed by the upper-middle bronchus emerged from an area between the right PA (A^1+3^) and the right superior pulmonary vein.

**Conclusion:**

The finding of A^4+5^ branching from the right main PA and descending posterior to the right upper-middle bronchus, which formed a common trunk, resembled a mirror image of the normal left lung. To our knowledge, a common trunk with the mediastinal type of the right PA has never been reported during video-assisted right middle lobectomy. In patients with heterotaxy syndrome, 3D-CT to preoperatively understand their anatomy is essential.

## Introduction

Anatomical abnormalities affecting pulmonary vessels have long aroused interest among thoracic surgeons, and numerous variations in the anatomy of pulmonary vessels have been reported [1, 2]. Indeed, in addition to fused interlobar fissure, calcified hilar adenopathy, and oncologic problems, vascular anomality is a crucial reason for converting from lobectomy to thoracotomy during video-assisted thoracic surgery (VATS) [1]. To preoperatively evaluate and classify pulmonary vascular drainage patterns, thin-section chest computed tomography (CT) [3] and three-dimensional (3D) CT [4–7] are potential tools. Notably, 3D-CT can be used to precisely determine the stereoscopic positional relationships among anatomical structures preoperatively.

Herein, we reported that an exceedingly rare case involving a patient in whom the upper and middle lobar bronchi formed a common trunk with the mediastinal type of the right pulmonary artery (PA). Using VATS, we were able to safely accomplish right middle lobectomy (RML) + systematic complete hilar and mediastinal lymph node dissection after employing 3D-CT to preoperatively consider this patient’s anatomical structure.

## Case presentation

**Fig. 1:**
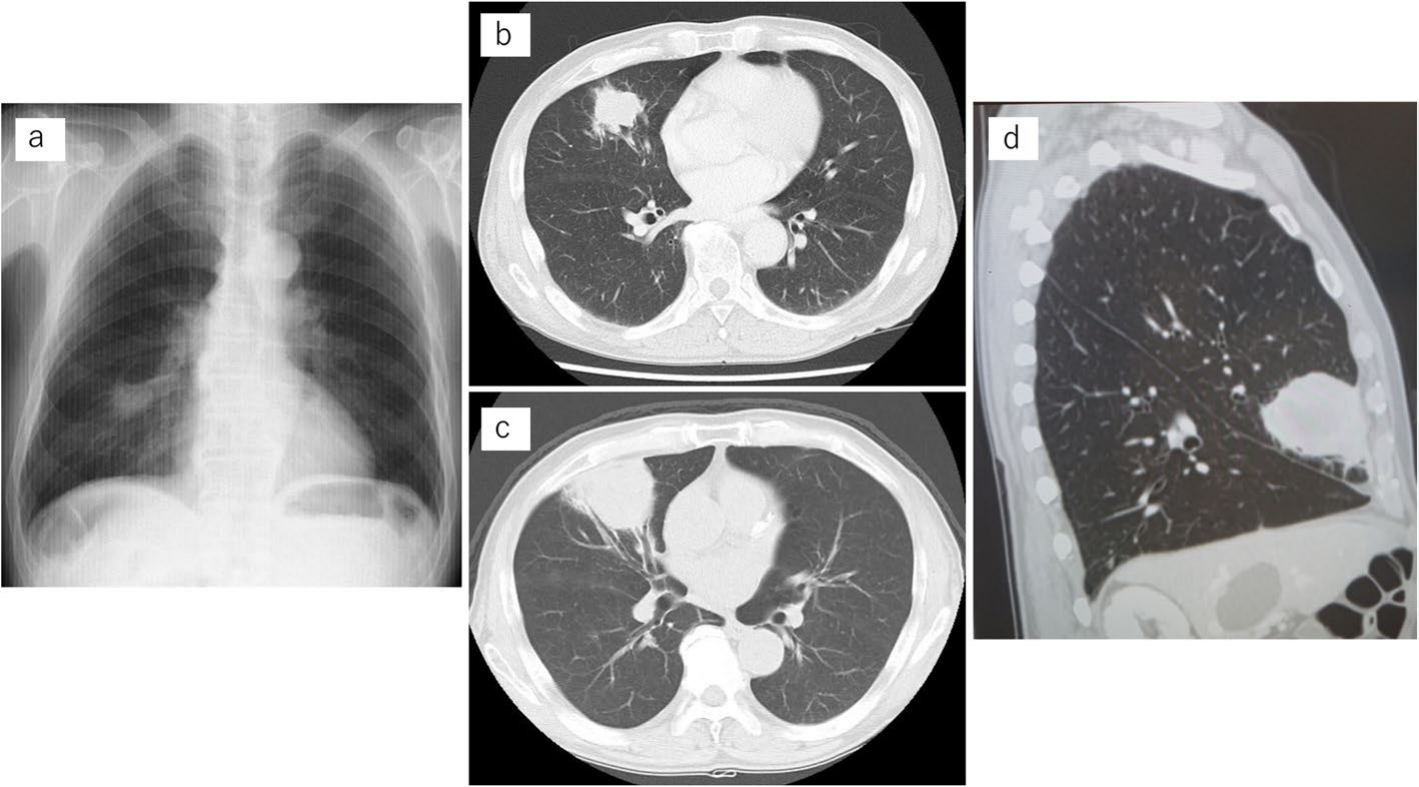
Chest X-ray showed a pulmonary nodule in the right lower lung field (**a**). Chest computed tomography (CT) revealed a pulmonary nodule 25 mm in diameter in the middle lobe of the right lung (**b**). Two months later, at the first visit to our University Hospital, CT revealed a rapidly growing tumor 60 mm in diameter (**c**). CT showed that the interlobar fissure between the right upper and middle lobes was incomplete (**d**)

**Fig. 2:**
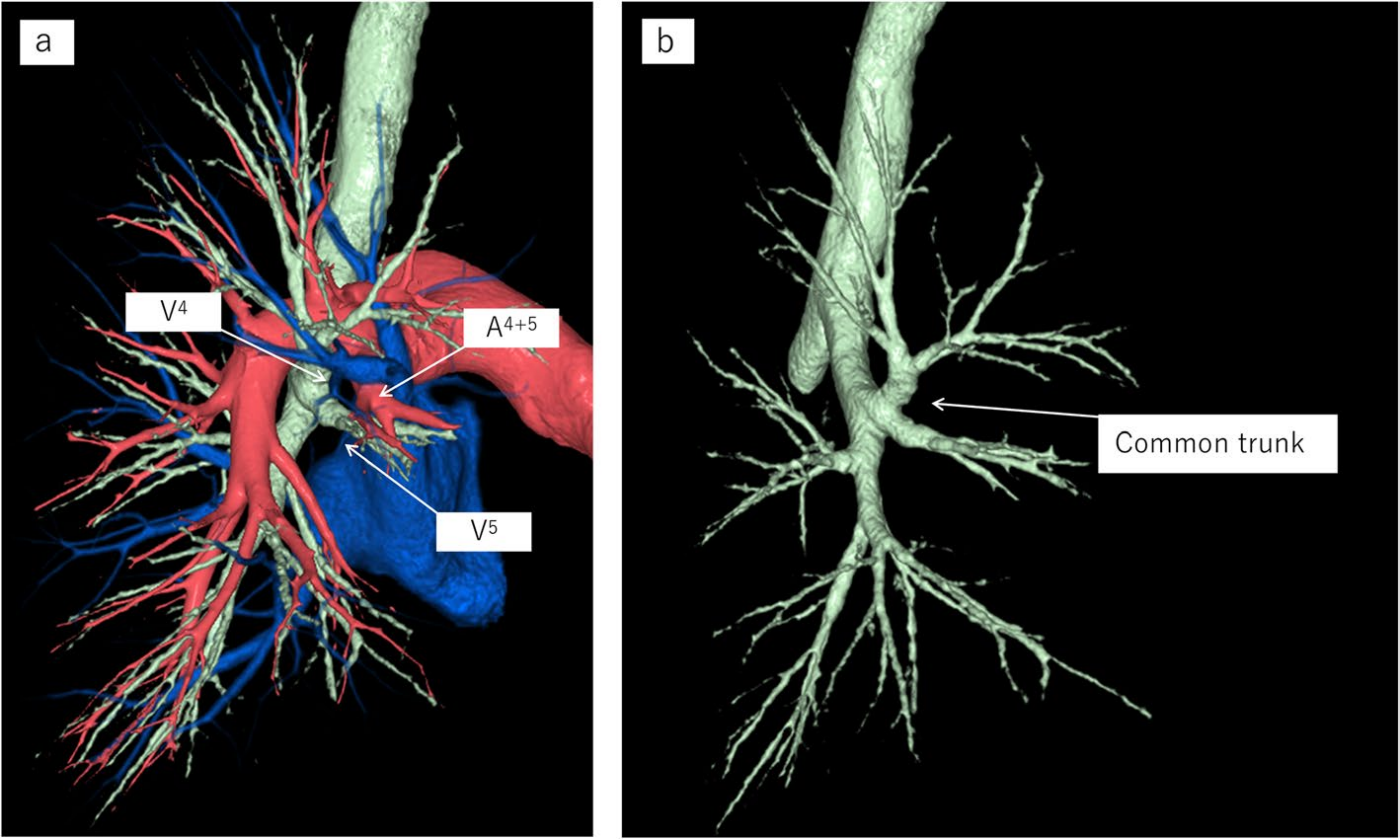
3D-CT revealed that the upper and middle lobar bronchi formed a common trunk with the mediastinal type of the right pulmonary artery. The arrows show the pulmonary artery and vein of the middle lobe. An anomalous A.^4+5^ was branched from the main pulmonary artery behind the superior pulmonary vein (**a**)

**Fig. 3:**
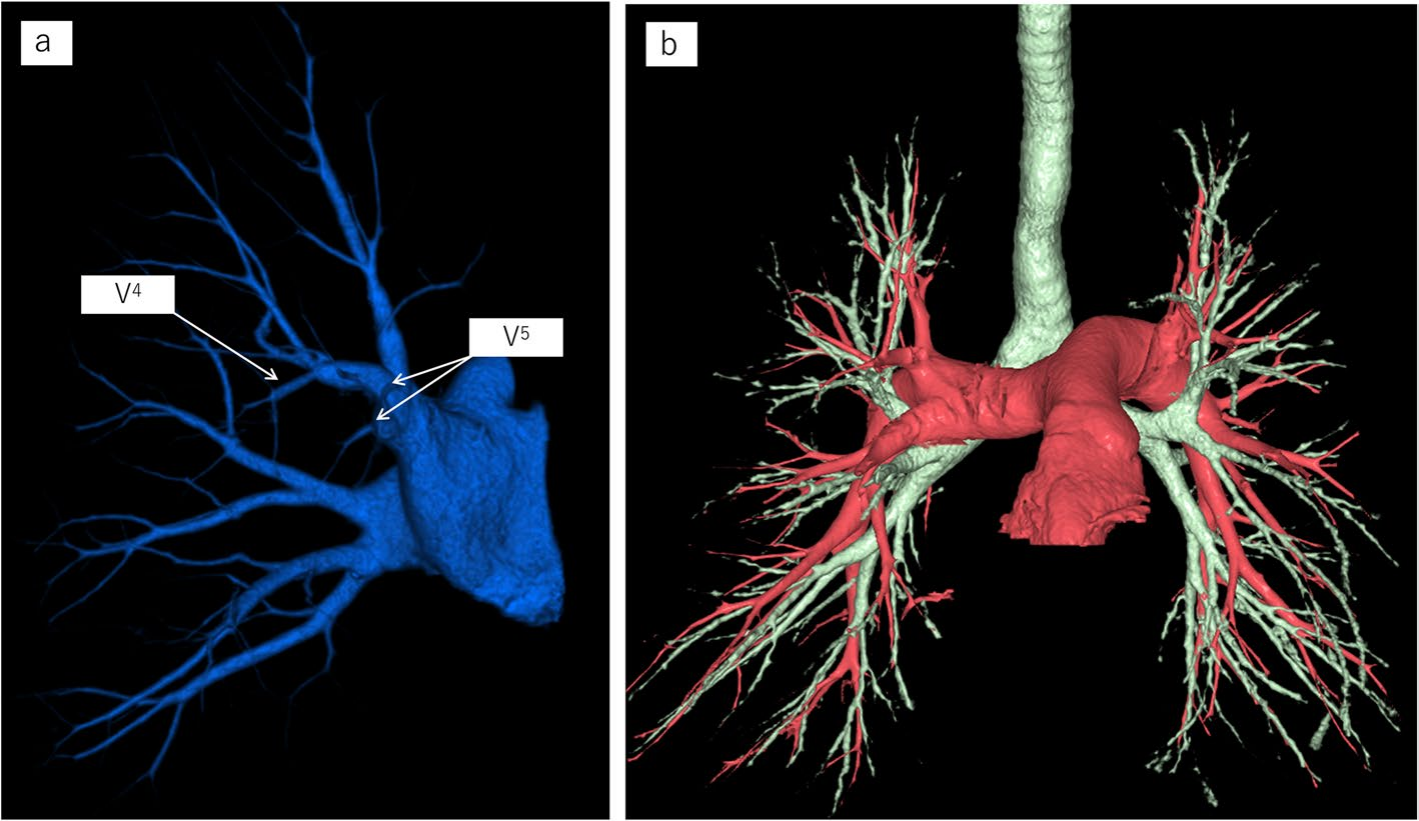
3D-CT revealed that V^4^ was connected to V^2^, and V.^5^ was independently draining into the superior pulmonary vein (**a**). The right PA is a common trunk with the mediastinal type of the right PA. The common trunk supplies the RML, which is similar to the mediastinal lingular artery of the left PA (**b**)

The patient was a 71-year-old man undergoing treatment for crowned dens syndrome and hypertension. In 2022, an abnormal shadow was detected in the right lung on X-ray during a health check-up at a nearby hospital (Fig. 1a). CT revealed an irregular nodule 25 mm in diameter in the right middle lobe, originating from the B5 bronchus and showing no swelling of the mediastinal or hilar lymph nodes (Fig. 1b). Based on subsequent bronchoscopy findings, lung adenocarcinoma originating from the right S5 was diagnosed. Two months later, at the first visit to our University Hospital, CT revealed a rapidly growing tumor that had reached 60 mm in diameter (Fig. 1c and d). On the basis of those findings, we diagnosed clinical T3(PL0)N0M0; clinical Stage IIB (8th edition TNM stage classification for lung cancer [8]). Using preoperative chest CT, bronchoangiographic 3D-CT images were constructed using a high-speed 3D-image analysis system (SYNAPSE VINCENT, Fujifilm Corporation, Tokyo, Japan). The 3D-CT revealed that the upper and middle lobar bronchi formed a common trunk with the mediastinal type of the right PA (Figs. 2a and b, 3a and b). No hemodynamic abnormalities or cardiac malformations were found.

**Fig. 4:**
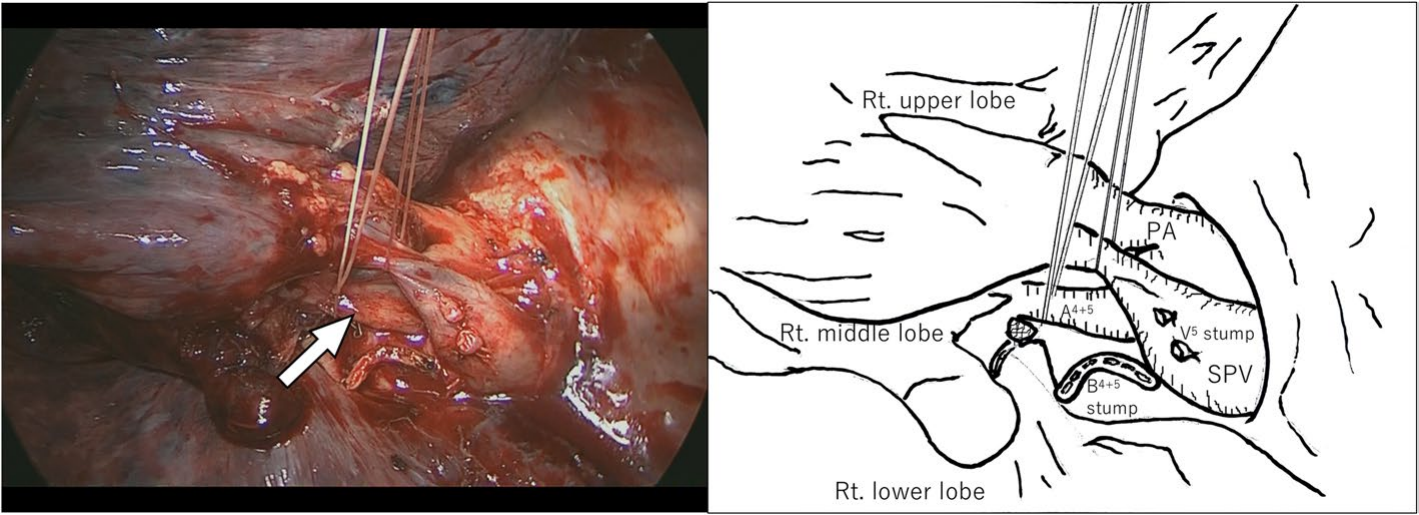
Intraoperative findings from the operation video (VATS RML). Captured photographs show an anomalous common trunk with the mediastinal type of the right pulmonary artery (white arrow)

The patient received the standard pre- and intraoperative care, which entailed curative resection by RML + ND2a-2 (and #3p sampling) under VATS with one-lung ventilation. The patient’s postoperative course was uneventful until discharge on the 7th postoperative day. Figure 4 (from the operation video) shows intraoperative findings of A^4+5^ branching from the right main PA and descending posterior to the right superior bronchus, which is similar to the tracheobronchial pattern of the left lung. The interlobar fissure between the right upper and middle lobes was incomplete. This minor fissure was divided/made using a stapler (Echelon Blue, 60 mm). After the superior pulmonary vein (SPV) was identified, V^4^ was connected to V^2^, and V5 was independently draining into the SPV. V^4^ and V^5^ were transected, respectively. However, an anomalous A^4+5^ directly branching from the main PA was discovered behind the SPV. This anomalous A^4+5^ was also cut after double ligation. The right middle bronchus was resected using a mechanical stapler (Echelon Gold, 60 mm) without covering the stump. The pathological diagnosis was keratinizing squamous cell carcinoma, total size 57 × 53 × 52 mm (= invasive size), pm0, pl0, ly1, v1, br (-), pT3, pN2, and pathological stage IIIB. Based on 22C3-immunohistochestry, PD-L1 expression was confirmed to be < 1%. Molecular testing was negative for EGFR and other oncogenes.

## Discussion

There is a great deal of anatomical variation within the PA branching pattern [9], and in patients with anatomically abnormal pulmonary structures, thoracic surgeries can carry a risk of bronchovascular injury and critical bleeding [10, 11]. Consequently, identification of the PA branches is an essential key to successful anatomic pulmonary lobectomy/segmentectomy.

In most cases, the main PA arises from the right ventricular outflow tract and courses posteriorly and superiorly to the left of and posterior to the aorta. After dividing into two lobar branches (the right and left PA), the right PA lies anterior and inferior to the right main stem bronchus [9]. However, the incidence of pulmonary vascular anomaly is reportedly 16.4% [12]. Nonetheless, bronchi forming a common trunk with the mediastinal type of the right PA is extremely rare. This patient’s right PA arches over the right mainstem bronchus and dives into the major fissure from the posterior aspect, coursing laterally to the right mainstem and right lower lobe bronchus. The first, and largest, branch of the right PA is a common trunk with the mediastinal type of the right PA. The common trunk supplies the RML, which is similar to the mediastinal lingular artery of the left PA. With this anatomy, a lobectomy/segmentectomy would be difficult to accomplish safely and has the potential for critical PA bleeding if surgeons do not have precise anatomical information preoperatively.

Heterotaxy syndrome is a congenital disorder in which typically asymmetrical organ systems develop symmetrically, accompanied by cardiovascular malformations and/or anatomical abnormalities defined as left–right axis abnormal arrangement of one or more organ systems among the thoracoabdominal organs, including the lungs [13, 14]. Heterotaxy syndrome was known as asplenia syndrome and polysplenia syndrome, and organ systems that originally showed asymmetrical development were thought to show symmetrical development. Subsequently, with the accumulation of cases, it has been reported that the morphology of the spleen, such as asplenia and polysplenia, does not necessarily reflect the structural morphology of the organ. This includes both disease groups, and it is not necessary for any of the atrium, thoracic organs, or abdominal organs to exhibit right-sided or left-sided structures; if at least one organ system shows a left–right differentiation disorder, it is considered a heterotaxy syndrome [15, 16]. This syndrome is classified as either right or left isomerism, depending on the morphology of the paired structures. Bronchial situs can be determined from the relationship between the upper lobe bronchus and the PA. Right isomerism is characterized by bilateral tri-lobed lungs and a bilateral upper bronchus branching superior to the level of the PA (an eparterial bronchus). Left isomerism is characterized by two bilobed lungs and a bilateral upper bronchus branching inferior to the level of the PA (a hyparterial bronchus) [17]. Other features such as the number of lobes, ratio of left to right main bronchial lengths, and branching pattern are less reliable markers [18]. The left and right pulmonary arteries do not need to be perfectly symmetrical to define heterotaxy syndrome. Patients with heterotaxy syndrome can also present with various cardiac malformations [19], which makes detailed cardiac evaluation before surgery indispensable for these patients. In addition, in the case of pulmonary resection, it is extremely important to check for abnormal pulmonary venous return, which can lead to fatal complications such as right ventricular failure. The present case had not been diagnosed prior to the initial consultation, but preoperative CT scans revealed heterotaxy syndrome, as the lungs were bilaterally bilobed with hyparterial bronchi. Fortunately, the cardiac function was good at EF 65.8%, the patient’s hemodynamics were normal, and no cardiac malformation was found. However, given that the patient’s right side anatomy resembled that of the left side and the tumor was located in the right middle lobe, right upper-middle lobectomy corresponding to left upper lobectomy may have been more favorable.

3D-CT is a reliable and noninvasive technique used in clinical practice for chest imaging in patients with lung cancer and provides important anatomical information before surgery [4, 11]. Notably, the identification rate of abnormal branches based on preoperative 3D-CT pulmonary angiography is reportedly 97.7%, compared to intraoperative findings [11]. Still, because 3D-CT misses PA branches less than 1.5 mm PA in diameter, it must be used carefully. We anticipate that use of digital techniques, including 3D-CT, will continue to become more widespread and evolve in the field of surgery.

## Conclusion

In summary, surgeons should be aware of the anatomical abnormality associated with heterotaxy syndrome, as its identification can obviate serious complications, including critical bleeding, in patients undergoing radical lobectomy/segmentectomy for lung cancer. 3D-CT can help us to preoperatively understand the stereoscopic positional relationships among anatomical structures.

## Abbreviations

VATS: Video-assisted thoracic surgery
CT: Computed tomography
3D: Three-dimensional
RML: Right middle lobectomy
ND: Lymph node dissection
POD: Postoperative day
PA: Pulmonary artery
SPV: Superior pulmonary vein
